# Eccentric exercise-induced delayed onset trunk muscle soreness alters high-density surface EMG-torque relationships and lumbar kinematics

**DOI:** 10.1038/s41598-024-69050-x

**Published:** 2024-08-10

**Authors:** Michail Arvanitidis, David Jiménez-Grande, Nadège Haouidji-Javaux, Deborah Falla, Eduardo Martinez-Valdes

**Affiliations:** Centre of Precision Rehabilitation for Spinal Pain (CPR Spine), School of Sport, Exercise and Rehabilitation Sciences, College of Life and Environmental Sciences, University of Birmingham, Birmingham, B15 2TT UK

**Keywords:** Delayed onset of muscle soreness, Exercise-induced muscle damage, Eccentric, Concentric, High-density surface EMG, Torque steadiness, Motor control, Neurophysiology

## Abstract

We aimed to assess high-density surface electromyography (HDsEMG)-torque relationships in the presence of delayed onset trunk muscle soreness (DOMS) and the effect of these relationships on torque steadiness (TS) and lumbar movement during concentric/eccentric submaximal trunk extension contractions. Twenty healthy individuals attended three laboratory sessions (24 h apart). HDsEMG signals were recorded unilaterally from the thoracolumbar erector spinae with two 64-electrode grids. HDsEMG-torque signal relationships were explored via coherence (0–5 Hz) and cross-correlation analyses. Principal component analysis was used for HDsEMG-data dimensionality reduction and improvement of HDsEMG-torque-based estimations. DOMS did not reduce either concentric or eccentric trunk extensor muscle strength. However, in the presence of DOMS, improved TS, alongside an altered HDsEMG-torque relationship and kinematic changes were observed, in a contraction-dependent manner. For eccentric trunk extension, improved TS was observed, with greater lumbar flexion movement and a reduction in δ-band HDsEMG-torque coherence and cross-correlation. For concentric trunk extensions, TS improvements were observed alongside reduced thoracolumbar sagittal movement. DOMS does not seem to impair the ability to control trunk muscle force, however, perceived soreness induced changes in lumbar movement and muscle recruitment strategies, which could alter motor performance if the exposure to pain is maintained in the long term.

## Introduction

Delayed-onset muscle soreness (DOMS) represents a form of ultrastructural muscle injury that typically emerges following intense, unfamiliar exercises, particularly those emphasizing eccentric contractions^1,2^. Clinical signs of DOMS, include reduced force production, painful movement limitations, stiffness, swelling, and dysfunction in adjacent joints^1^. These symptoms can persistently impair muscle function^3^. Although considered a mild form of injury, DOMS frequently undermines athletic performance^1^ and may disrupt daily activities^3^, likely due to alterations in electromyography (EMG)-force relationships, during muscle contractions^3^. Evaluating the relationship between EMG and force/torque provides valuable insights into neuromuscular function^4^. EMG measures the electrical activity of muscle fibers, indicating the efficiency of neuromuscular transmission, which is the process by which the nervous system activates muscles to generate force^4,5^. This electrical activity must be efficiently translated into mechanical output for effective muscle function. During DOMS, neuromuscular function may be compromised, potentially affecting this translation process. Assessing EMG-torque relationships can help us uncover the underlying mechanisms responsible for alterations in motor function.

Numerous studies have investigated the effect of DOMS on the neuromuscular function of peripheral muscles, such as the elbow flexors and hamstrings^3,6–11^. In contrast, research focusing on trunk muscles is less extensive, with only a few studies inducing DOMS to assess its influence on trunk muscle function. For example some of these investigations have revealed altered activation patterns^12,13^ and decreased force accuracy^2^. Given these findings and considering that people often move differently when experiencing pain^14^, it is hypothesized that DOMS may also lead to changes in trunk movement patterns, yet this aspect remains poorly understood. Additionally, it remains to be investigated whether DOMS alters trunk extensor muscle torque steadiness.

Performing contractions with minimal force fluctuations is crucial in everyday life activities, as reduced torque steadiness can affect the precision of voluntary movements and functional ability. During a voluntary contraction, the output from the activated motor unit population leads to force generation, which is not constant but rather fluctuates around an average value^15^. Years of investigation into the neural mechanisms causing variations in muscle force during voluntary contractions have revealed that the neural command's low-frequency component (< 10 Hz) is the most relevant for force generation^16^. This component mirrors the common synaptic input received by the motor unit population^17^. It has previously been demonstrated that eccentric exercise can cause increased elbow flexor muscle force fluctuations, likely due to an increased common neural drive to the muscles^18^. Given these findings, it is relevant to explore the effect of DOMS on torque steadiness, trunk muscle activation, and spine kinematics during dynamic contractions.

To understand the relationship between muscle activity and the force output, surface EMG (sEMG)/force relationships have been extensively examined. Such investigations^19,20^ have identified an association between low-frequency force fluctuations and the corresponding low-frequency component of the rectified interference sEMG. For certain muscles such as the erector spinae (ES), where HDsEMG decomposition is challenging, this methodology offers an alternative, especially when establishing the correlation between motor unit discharge times and force (the gold standard) is difficult. Staudenmann et al.^21,22^ have indicated that employing high-density sEMG (HDsEMG), known for its superior spatial sampling resolution, alongside the application of Principal Component Analysis (PCA), enhances the force estimations based on sEMG. PCA acts as a dimensionality-reduction tool that can identify and select a subset of principal components (PCs) accountable for the majority of data variation, thereby explaining most of the variance in the exerted torque^21^. This method has been applied previously to quantify both magnitude and regional alterations in HDsEMG-torque relationships in individuals with and without chronic low back pain (CLBP) during isometric^23^ and dynamic trunk extension^24^. However, how these relationships are altered in the presence of thoracolumbar ES DOMS remains unclear. Given the frequent use of trunk muscles in daily activities involving eccentric contractions, understanding the effects of DOMS on these muscles is crucial. Specifically, changes in trunk neuromuscular function, such as increased force fluctuations and altered recruitment strategies, may predispose individuals to more severe muscle injuries under certain conditions^25^.

This study aimed to explore motor adaptations and changes in recruitment strategies due to DOMS by evaluating its influence on various motor performance measures and HDsEMG parameters measured from the thoracolumbar ES during both concentric and eccentric trunk extension contractions. Specifically, the primary objectives were to (i) quantify changes in the relationship between HDsEMG oscillations and torque oscillations in both time and frequency domains, (ii) assess and compare regional differences in HDsEMG amplitude and HDsEMG–torque cross-correlation and coherence of the ES, and (iii) investigate differences in torque steadiness and associated kinematic data from the lumbar spine following the induction of DOMS in asymptomatic individuals. It was hypothesized that individuals would exhibit reduced torque steadiness under the influence of DOMS, accompanied by alterations in EMG-torque relationships and lumbar kinematics. Secondary objectives also included assessing changes in muscle soreness, thoracolumbar sensitivity measured as pressure pain thresholds (PPTs), and muscle strength measured as peak torque over three days to confirm the presence and progression of DOMS.

## Methods

### Study design and setting

This experimental study with a repeated measures design received approval from the Ethical Review Committee at the University of Birmingham, United Kingdom (approval number: ERN 19–1148) and adhered to the Declaration of Helsinki. Data collection spanned from April 2019 to July 2022 at a laboratory within the Centre of Precision Rehabilitation for Spinal Pain, University of Birmingham, United Kingdom. The study was conducted over three sessions on consecutive days, and each were separated by approximately 24 h (baseline, post 24 h, post 48 h). These time points were selected similarly to previous research^3^ to capture the progression of DOMS, considering that its onset typically occurs at 24 h and peaks within 72 hours^26,27^. At baseline, the measurements taken immediately after the eccentric exercise protocol (described later) were conducted to confirm that the protocol was effective in inducing immediate muscle soreness^28^. All participants gave their written consent before involvement.

### Participants

Twenty asymptomatic controls (ten males, ten females) were recruited from the local Birmingham community, including the University of Birmingham's student and staff populations, through social media announcements and distributed information leaflets. The sample size was based on previous studies^12,13^ that used a similar number of participants but also on a moderate effect size of *f* = 0.36, an *α* of 0.05, a *β* power of 0.9 and a 10% data loss due to signal quality of participant withdrawal, for a repeated measures analysis of variance (ANOVA) using a within factors design consisting of three measurement points. The effect size was calculated from the mean ± SD of lumbar ES HDsEMG reflex amplitude values before and after the induction of DOMS (averaged effect sizes for both sides and first and last trials) reported by Abboud et al. (2021)^12^.

Participants were eligible for the study if they were men or women aged 18–55 years, asymptomatic, with no prior back or lower limb pain requiring medical attention. Exclusion criteria included cardiovascular diseases, pregnancy, spinal deformities or surgeries, systemic or inflammatory conditions, rheumatic and neuromuscular disorders, neurological conditions, and lumbar radiculopathy. During the three days, as well as the day preceding the experiment, participants were advised against engaging in any high-intensity or atypical exercises and refraining from taking medications intended to alleviate pain or soreness.

### Questionnaires

At the start of the session, to assess the level of physical activity, participants were asked to complete the full version of the International Physical Activity Questionnaire (IPAQ). The reliability and validity of the complete IPAQ have been previously established^29^. Lumbar muscle soreness was also verbally assessed at the beginning and end (i.e., immediately after the eccentric exercise protocol) of the first session. Subsequent evaluations were made at the beginning of the following sessions at 24 and 48 h, respectively. Participants were instructed to evaluate the subjective intensity of movement-related lumbar muscle soreness using an adapted 0–10 visual analogue scale (VAS), where 0 represented "no soreness at all" and 10 signified "extreme soreness"^8,30^. During the eccentric exercise protocol (detailed below), participants were prompted to indicate their perceived exertion levels (after each set of 5 repetitions) using a modified Borg scale (Category-Ratio-10 Scale; Borg, 1998). The scale spans from "0" (no perceived exertion) to "10" (extreme exertion).

### Pain sensitivity assessment

PPT testing was performed to quantitatively assess changes in thoracolumbar ES sensitivity across consecutive days after the induction of DOMS and confirm local sensitisation. Following a familiarisation phase, PPTs were measured with the participants lying on a plinth in a prone position, using an electronic algometer (probe tip: 1 cm^2^, 30 kPa/s; NOD, OT Bioelettronica, Italy). Participants were guided to indicate to the researcher when the sensation of pressure transitioned to pain, at which moment the pressure application ceased.

PPTs were performed only on the right side for all participants over ten testing sites (spanning approximately from L5 to T10)^24^. Specifically, two vertical rows were marked, each consisting of 5 PPT sites. These rows started from the L5 vertebra and extended vertically to approximately the T10 vertebra, covering a total distance of approximately 20 cm. The five PPT sites were spaced equally along this distance (approximately 4.8 cm apart). The medial row was positioned 2 cm lateral from the spinous processes over the ES muscles, while the other row was placed 3.2 cm further lateral. At each location, two PPT measurements were performed (randomised order), and the average of the two measurements at each site was used for subsequent analysis (mean thoracolumbar ES sensitivity). PPTs were assessed at the beginning and end of session 1 (immediately after the eccentric exercise protocol), as well as at the start of session 2 (24 h later) and session 3 (48 h later). Topographical maps of PPTs were also generated using the mean values of all 10 PPT sites across all participants. The centroid (x-axis and y-axis coordinates) was computed to identify the region of increased sensitivity (i.e., areas with the lowest PPTs). This approach facilitated comparisons of sensitivity areas across different days and was also useful to understand if the location of pain was related to regional changes in HDsEMG-based parameters (e.g., muscle activity, coherence) mentioned below. The same researcher performed all PPT measurements, ensuring the reduction of inter-experimenter variability.

### Electromyography

Surface HDsEMG recordings were acquired in monopolar configuration using two 2D electrode grids, each arranged in a 13 × 5 pattern. These grids had evenly spaced electrodes (diameter: 1 mm, spacing: 8 mm; GR08MM1305, OT Bioelettronica, Italy), with one electrode missing in the upper left corner. Each HDsEMG electrode was attached with a double-sided adhesive foam (FOA08MM1305, OT Bioelettronica, Italy). Conductive gel was then applied to the electrode sections of the grids to ensure proper skin contact (AC-CREAM, SPES Medica, Genoa, Italy). Participant skin preparation included shaving (if required), mild abrasion using an abrasive gel (Nuprep Skin Prep Gel, Weaver and Company, Aurora, Colorado), followed by water rinse and drying. To ensure consistent electrode placement across the three days, the electrode placement area was marked with a surgical skin marker. Additionally, room temperature was controlled to maintain a stable environment. To monitor thoracolumbar ES activity, the two HDsEMG grids were placed vertically on the right side, starting 2 cm lateral to the spinous process of the 5th lumbar vertebra and extending up to approximately the 10th thoracic vertebra, for all participants^24,31^. Reference electrodes (WhiteSensor WS, Ambu A/S, Ballerup, Denmark) were also affixed to the participant's sacrum, the anterior superior iliac spine (ASIS), and wrist. For a comprehensive description and visual representation of the HDsEMG electrode placement, we direct readers to our previous work^24^.

Both torque and HDsEMG signals were sampled at a rate of 2048 Hz. These signals were digitised using a 16-bit A/D converter (Quattrocento, 400-channel EMG amplifier, OT Bioelettronica, Torino, Italy, amplification: 150, frequency range 10–500 Hz, first order, 3 dB). The OTBiolab + software platform was used for data acquisition.

### Lumbar kinematics

Lumbar movements during the contractions were quantified using Noraxon's myoMOTION system and two wearable Inertial Measurement Units (Research PRO IMUs, Noraxon USA) with a sampling rate of 100 Hz. The sensors were attached via double-sided tape on the lower thoracic and lumbar spine (T12 and L5, respectively). Using Noraxon's myoRESEARCH software (version 3.14), custom angles were created based on the difference between these two sensors. This allowed lumbar flexion/extension, lateral flexion and rotation angles to be captured during all contractions. Before any measurement commenced, participants were asked to adopt a natural upright position while sitting on the dynamometer's chair, and the sensors were calibrated. The myoMOTION software and hardware allowed the synchronization of the myoMOTION receiver with other systems.

### Isokinetic dynamometry

An isokinetic dynamometer (System 3 Pro, Biodex Medical Systems, New York) was utilised to assess the torque produced by participants during concentric and eccentric trunk extension maximal voluntary contractions (MVCs) and the torque steadiness tasks performed at submaximal levels. To focus on the lumbar spine, participants were positioned on the Biodex Dual Position Back Extension/Flexion Attachment, ensuring their hips and knees were at a 90° angle and feet spaced at shoulder width^23,32^. The chair's seat was tilted upwards approximately 15°, and its height was adjusted so the dynamometer's rotational axis aligned with the ASIS bilaterally^32^. To prevent compensatory movements, participants' upper trunk, pelvis, and thighs were securely strapped to the chair. A specific Biodex attachment was also in place to reduce knee muscle engagement. This attachment included pads placed on both the anterior surfaces of the tibiae at the proximal level.

For all contractions, the dynamometer operated in isokinetic mode. The range of motion (ROM) for both concentric/eccentric extension contractions was 50°, spanning from 20° extension to 30° flexion, emphasising lumbar movement and minimising compensatory actions from the legs^32^. It should be noted that these angles refer to the attachment angles rather than thoracolumbar angles. Therefore, participants had some freedom of movement and could alter their lumbar kinematics by utilizing a more stooped position, performing pelvic tilts, or lateral bending and slight rotation, which could be captured with kinematic analysis. A consistent angular speed of 5°/s was maintained. When returning to the starting position, the concentric contraction mode was consistently set at an angular velocity of 90°/s. For the eccentric mode, the return velocity was adjusted to 20°/s to ensure optimised comfort for the participant. Nonetheless, participants were assisted back to the initial position by the researcher, who manually readjusted the chair for every repetition.

### Eccentric exercise protocol (DOMS)

The eccentric exercise protocol consisted of three sets of 15 eccentric contractions of the trunk extensors, moving from 20° of trunk extension to 30° of trunk flexion. The protocol was performed only at the end of session 1. Resistance was set at 50% of their MVC (the %MVC was chosen after pilot testing to induce mild-moderate muscle soreness), with an angular speed of 5°/s. Participants were given a 30-s rest between each set. Participants were asked to perform an MVC immediately after the eccentric contractions to further assess the trunk extensors and validate the effectiveness of the eccentric exercise to induce fatigue (i.e., reduction in maximal torque output).

### Experimental protocol

Participants were given time to familiarize themselves with the dynamometer, by practising all contractions and warming up their trunk extensor muscles via sustained and dynamic submaximal contractions.

After a brief rest, they were asked to perform two concentric trunk extension MVCs, moving through a 50° ROM. The starting position for the concentric trunk extension, MVC, was 30° of trunk flexion, and the final position was 20^o^ of trunk extension. Between these contractions, 1-min rest was provided and each lasted 5 s. After a 5-min rest, participants were instructed to perform submaximal concentric trunk contractions, four times at 25% and three times at 50% MVC, in a randomised order. During these contractions, they were asked to match the target torque lines at 25% and 50% MVC, keeping the contraction for 10 s. They were allowed to practice each submaximal contraction once before the actual recordings. The highest peak torque observed during the MVC was used to set the submaximal torque targets. After these, participants repeated the same procedures for the eccentric trunk contractions (i.e., the concentric and eccentric contractions were performed separately). The only difference for the eccentric trunk extension contractions was that the starting position was at 20° of trunk extension, and the final position at 30°of trunk flexion (i.e., the opposite from the concentric contractions).

**Figure 1:**
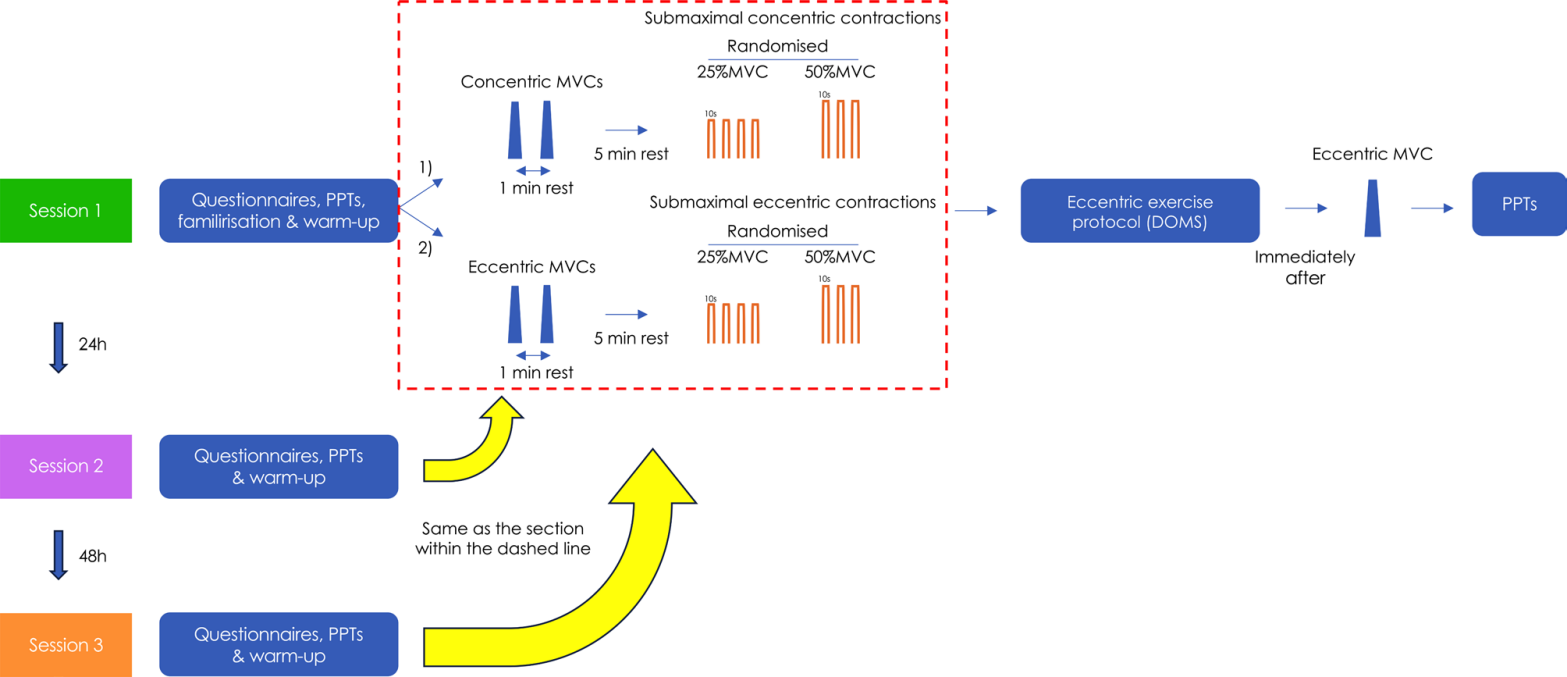
Schematic representation of the overall study procedure. Participants performed the same concentric and eccentric contractions 24 h and 48 h after the induction of delayed onset trunk extensor muscle soreness (part within the red dashed line).

Throughout all submaximal contractions, real-time visual feedback of their torque output and a line representing the desired contraction level (%MVC) was visible on a computer screen positioned 1.5 m in front of them. This real-time torque data was superimposed over the template for visual feedback. Participants aimed to swiftly and accurately match the %MVC target. Once they achieved the set target (either 25% or 50% MVC), they were advised to keep their torque output as steady as possible for the whole contraction. Essentially, they were asked to produce consistent torque output (25% or 50%MVC) across the entire 50° ROM. The overall study procedure is depicted schematically in Fig. 1.

#### Analysis of the torque signal

The highest peak torque during the MVCs was used to assess each individual's maximum trunk extension concentric and eccentric strength. Torque steadiness was evaluated by calculating the absolute and relative amplitude of the torque fluctuations, determined by the standard deviation and the CoV of torque, respectively (CoV, standard deviation of the torque/mean of torque × 100)^15^. A custom MATLAB script was employed to allow the computation of the SD and CoV of torque within the same time window used for the HDsEMG analysis, during the steady phase of the contraction. These values were determined for each repetition and then averaged to provide a single value for each torque level for each contraction. The time windows selected for analysis were approximately 8 s for both submaximal torque levels. This approach aimed to exclude the first and last second of the steady part of the contraction, periods during which participants typically overestimated or underestimated the requested torque level.

#### EMG amplitude and topographical map computations

During the offline analysis, the 128-monopolar channels from the merged electrodes over the thoracolumbar ES, were processed to obtain 124 bipolar channels. These were obtained by differentiating the monopolar HDsEMG signals in the presumed vertical direction of the muscle fibers (i.e., in the direction of the electrode columns). Before the root mean square (RMS) amplitude calculations, the HDsEMG signals were filtered with a bandpass zero-lag Butterworth filter (10-350 Hz, 2nd order) and visually inspected, to exclude channels with low-quality signals due to electrical interference and/or artifacts. Less than 15% of the channels were discarded (per electrode grid).

One RMS value was calculated for each of the 124 bipolar channels for the thoracolumbar ES during all trunk eccentric/concentric extension contraction. By determining the RMS for each channel, a HDsEMG amplitude map was generated for the (agonist) thoracolumbar ES and the centroid (x-, y- coordinates) of this map was calculated using all channels, during all muscle contractions. This allowed us monitor regional changes in thoracolumbar ES activation. Additionally, a global measure of myoelectric activity was calculated for each muscle, by calculating the average of RMS values from all channels for each muscle, which formed a single value (HDsEMG amplitude; RMSmean). In this study, EMG signals were not normalized due to the established reliability of HDsEMG-derived measures in within-subject designs, particularly during voluntary trunk movements^33^.

All HDsEMG and torque data was assessed offline using a custom MATLAB 2020b script (The MathWorks Inc., USA). Concentric and eccentric muscle contractions were recorded separately.

#### HDsEMG data pre-processing for PCA analysis

PCA is a dimensionality reduction technique that effectively identifies redundant information in HDsEMG data. This approach can also enhance the accuracy of HDsEMG-based force estimations^21,22^. By transforming complex, multivariate data into a series of linearly independent PCs through an orthogonal transformation, PCA reduces dimensionality while retaining most of the data’s variance. In this study, these components, which were linear combinations of the original 124 channels, were organized in descending order based on the variance they accounted for, with the first few components representing the majority of the data's variance. PCs that collectively accounted for a cumulative 85% of the total variance were retained^23,24,34^ to keep only the most informative components and improve the signal-to-noise ratio. This process, which marked the initial phase of dimensionality reduction, generated a new matrix containing the eigenvectors of these selected PCs. The original dataset, therefore, remained intact until the transformation in the final phase of PCA. In this phase, the dataset was reorientated from its original axes to those delineated by the selected PCs. This was achieved by transforming the original dataset into a lower-dimensional space (i.e., < 124) precisely defined by these PCs that explained 85% of the variance in the data. Consequently, the original 124-channel dataset was transformed into a new dataset with reduced dimensionality that explained most of the variance in the data. For further details on the PCA methodology, please refer to earlier work^23,24^.

Before performing the cross-correlation and coherence analyses, the formerly offline-referenced 124 bipolar HDsEMG channels (from the combined electrodes placed over ES) underwent the following pre-processing for PCA computations: (1) 10 Hz high-pass filtering, (2) selection of the most informative subset of PCs using PCA on the 124 (for ES) differential HDsEMG signals in temporal domain, (3) full-wave rectification and averaging the selected PCs to generate a single time signal for the thoracolumbar ES muscle, (4) low-pass filtering at 10 Hz (supplementing the initial filtering), aiming to detect slow-frequency variations in motor unit activation/recruitment^16^, (5) application of a first-order Savitzky-Golay filter for smoothing, and (6) removal of DC (zero frequency) components^21–24^. These processing procedures resulted in a final signal envelope that was obtained by applying PCA to the HDsEMG grid and contained the low-frequency components of the HDsEMG data.

Coherence and cross-correlation analyses were then conducted to estimate the similarity between this final EMG signal envelope and torque signals. Please note that the PCA and subsequent coherence and cross-correlation analyses were exclusively conducted for the thoracolumbar ES, which functioned as the primary agonist muscle during the eccentric and concentric contractions.

#### Cross-correlation and coherence analyses

Cross-correlation analysis quantified the similarity (cross-correlation coefficient) between the final signal envelope (specifically, the low-pass filtered average PCA-selected signal derived from the first PCs accounting for 85% variance) and torque signals in the time domain. The relationship between torque and EMG was further investigated through coherence analysis, as done previously^23^.

Coherence analysis, aiming to measure the intensity and frequency of synchronous synaptic inputs across the motor unit population in relation to torque, was executed using the magnitude squared coherence (MSC) method with a 1-s Hamming window and 50% overlap, as detailed earlier^35^. Given the contraction's brief duration, this method was chosen to optimize analysis resolution while minimizing data loss. The same sEMG envelope (Final Signal Envelope) was evaluated against the torque signal in the frequency domain. MSC, a frequent measure to evaluate signal similarity in the frequency domain, was computed using MATLAB's *mscohere* function. This function applies Welch's overlapped averaged periodogram method with a 50% overlap across N sub-windows. Further MSC computation insights can be found in previous studies^23,36^. The Fisher's z-transformation was utilised on coherence estimates (C) to enable statistical comparisons, as these transformed values (FZ) follow a normal distribution. Given the potential crosstalk affecting sEMG recordings, bias was determined as the coherence profile's peak value at 250 Hz, an area with no significant correlated activity^35^. The utilised equation was the following:

$$FZ = {\text{atanh}}\left( {\sqrt C } \right) - bias$$

This study's coherence analysis focused on the δ band (0–5 Hz), which is most relevant to muscle torque generation^17^. Additionally, topographical coherence maps, as described in previous research^23^, were generated. In this process, each of the 124 HDsEMG signals from the thoracolumbar ES was individually assessed for coherence in frequency with the filtered torque signal. This analysis resulted in 124 distinct coherence values, one for each HDsEMG signal. These values were then utilized to construct topographical coherence maps. The maps were then normalized to the maximum coherence value at each torque level. The coherence's centroid provided an estimate of the centre of ES muscle δ-band coherence along both medial–lateral (x-axis) and cranial-caudal (y-axis) directions. This analysis enabled us to examine if certain areas of the thoracolumbar ES have greater influence on torque generation and to determine whether the regions with predominant influence shifted across days due to muscle soreness, by statistically comparing x- and y-axis centroid values across days. Previous studies^23,24^ have shown that individuals with CLBP exhibit regional differences in HDsEMG-torque coherence compared to pain-free individuals, leading us to determine if similar regional differences occur in the presence of DOMS.

**Figure 2:**
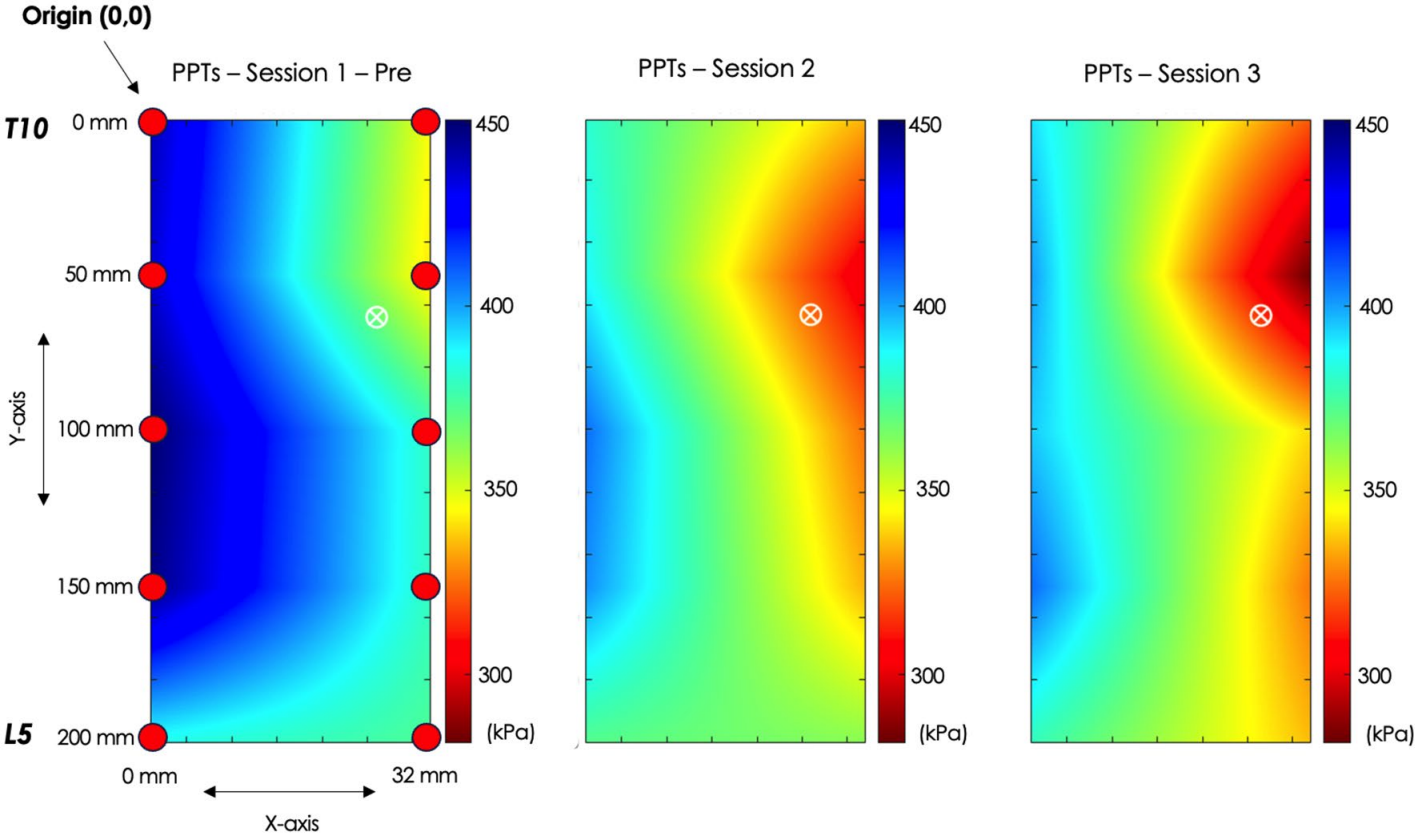
Topographical maps of PPTs are presented across three consecutive days: baseline (session 1-pre), after 24 h (session 2), and after 48 h (session 3). The red coloration indicates areas of heightened sensitivity, while blue colours represent areas of less sensitivity, denoting higher PPT values. Notably, there is increased sensitivity observed at 24 h and 48 h compared to baseline. All PPT maps utilise the same scale and have been normalised according to the maximum and minimum of all values. The red circles show the PPT sites over the thoracolumbar area that spanned from T10 to L5 vertebrae, with the medial part of all maps being close to the spine. A white circle with an “X” inside represents the center of sensitivity, indicating the location of overall heightened sensitivity, which was similar during all days. The distances along the x and y axis are the coordinates of the centre of sensitivity and could also help understand the distance between the PPT points along the two axes. The origin for these coordinates is at the top left corner.

For all maps (i.e., RMS amplitude, coherence and PPT) the origin of the coordinates was at the top left corner of the grid, as depicted in the PPT maps in Fig. 2.

#### Analysis of lumbar kinematics

Custom anatomical angles (flexion/extension, rotation, and lateral flexion) were generated based on the differences between the two IMU sensors (T12 and L5). The raw data of custom angles was exported in MATLAB (R2020b, MathWorks) for further offline analysis. A low-pass Butterworth filter with a cut-off frequency of 10 Hz was applied to all kinematic data^37^. Kinematic angles were calculated for all contractions, for all days, during each repetition (25%: 4 repetitions, 50%: 3 repetitions) and then averaged. Based on the averaged data (average of all repetitions), eight epochs were created. The eight epochs were normalized by subtracting epoch 1 from all epochs and were converted to positive (absolute) values. This was necessary to allow the calculation of the area under the curve (AUC) for all movement planes (i.e., sagittal, frontal and transverse), which was used to assess differences in the magnitude of movement across days.

Please note that HDsEMG, torque, cross-correlation, coherence and kinematic analyses were performed separately for each type of contraction (concentric, eccentric). Additionally, for every repetition, a single value was computed, i.e., four values for 25%MVC and three values for 50%MVC. These were then averaged to form one value for each submaximal level. This analysis was consistently applied to the data from all three sessions and was used for the statistical analysis.

#### Statistical procedures

Statistical analyses were performed on SPSS Statistics, version 29 (IBM, USA). The Shapiro–Wilk and the Levene tests were used to confirm data normality and ensure homogeneity of variance, respectively. Given that these conditions were satisfied, parametric tests were used. Data are presented as mean and standard deviation (SD), unless otherwise specified. For the pre-and-post assessments of muscle soreness, PPTs and MVCs, paired samples *t*-Tests were employed.

For HDsEMG, torque and kinematic variables (i.e., RMS, centroid values of the RMS amplitude map in both axes, δ band sEMG-torque coherence, sEMG-torque cross-correlation, centroid values for sEMG-torque coherence in both axes mean torque, torque CoV, torque SD, AUC), a two-way repeated measures ANOVA was performed separately for each type of contraction (eccentric, concentric). The torque target (20%MVC and 50%MVC) and session (baseline, 24 h, 48 h) were used as within-subject factors. One-way repeated measures ANOVA was also used to monitor changes in muscle soreness, PPTs and MVCs across the three testing days.

In instances where the ANOVA indicated significant interactions, a Bonferroni correction was applied for detailed pairwise comparisons. The threshold for statistical significance was set at *p* = *0.05*.

## Results

### Muscle soreness

All 20 participants (10 males, 10 females) completed the study, with their characteristics detailed in Table 1. The eccentric exercise protocol performed at the end of session one induced a moderate level of movement-related back muscle soreness immediately post-exercise (4.1/10) (*t* =  *− 8.305, p* < *0.001*). Participants reported mild muscle soreness 24 h and 48 h compared to baseline (session effect: *F* = *28.222*, *p* < *0.001, ηp*^*2*^ = *0.744*). Muscle soreness immediately post-exercise was higher compared to that observed at 24 h and was similar to that observed at 48 h (session effect: *F* = *5.760*, *p* = *0.012*, *ηp*^*2*^ = *0.233*). Mean ± SD values of muscle soreness are reported in Table 1.

### Mean thoracolumbar PPTs

During the first session, after the eccentric exercise protocol, PPTs assessed over the lumbar region decreased significantly (*t* = *2.195*, *p* = *0.041*). This heightened mean thoracolumbar sensitivity (i.e., lower values of PPTs) persisted 24 h and 48 h post-baseline (session effect: *F* = *6.355, p* = *0.004*). No significant differences in thoracolumbar sensitivity were observed between measurements taken immediately after the eccentric exercise protocol, and those taken at 24 h and 48 h later (*F* = *0.744*, *p* = *0.482*). Mean ± SD PPT values are reported in Table 1.

**Table 1:** Demographic characteristics and descriptive data of all 20 asymptomatic individuals that participated in the study.

Characteristic	Mean ± SD
Age (years)	28.7 ± 3.9
Height (cm)	171.9 ± 7.4
Body mass (Kg)	70.6 ± 13.4
Body mass Index (kg/m^2^)	23.9 ± 4.2
PPT session 1–baseline (kPa)	397.8 ± 95.0
PPT session 1–post	370 ± 110.74
PPT 24 h	361.0 ± 113.7
PPT 48 h	357.9 ± 106.6
VAS session 1–baseline (0–10)	0.0 ± 0.0
VAS session 1–post	4.1 ± 2.2
VAS 24 h	2.6 ± 2.1
VAS 48 h	2.9 ± 1.8
IPAQ (total METs score)	4325.1 ± 3578.2
ECC MVC session 1–baseline (N m)	203.4 ± 48.9
ECC MVC session 1–post	174.2 ± 53.3
ECC MVC 24 h	194.8 ± 51.6
ECC MVC 48 h	191.4 ± 54.9
CON MVC session 1 (N m)	211.8 ± 58.7
CON MVC 24 h	200.2 ± 43.1
CON MVC 48 h	197.0 ± 48.1

Data are presented across different time points (baseline, after the eccentric exercise protocol, after 24 and 48 h respectively).

*PPT* pressure pain threshold, *VAS* visual analogue scale, *IPAQ* international physical activity questionnaire, *ECC* eccentric, *MVC* voluntary contractions, *METs* metabolic equivalents, *CON* concentric.

### PPT maps

PPT maps were generated to determine if regional pain sensitivity at 24 h and 48 h differed significantly from baseline in specific thoracolumbar areas due to the eccentric exercise protocol. There were no differences for both the y- and x-axes compared to baseline (session effect: *F* = *1.054, p* = *0.358, ηp*^*2*^ = *0.053* and *F* = *1.340, p* = *0.274, ηp*^*2*^ = *0.066* respectively). The PPT maps are illustrated in Fig. 2.

### Rate of perceived exertion

At the end of session 1, during the eccentric exercise protocol, the participants were asked to report their rate of perceived exertion on a modified Borg scale (10 point scale), every 5 repetitions. An increase in the rate of perceived exertion was observed, with the average Borg value at the end of the protocol being 7.4/10, suggesting that the task was interpreted as “very hard” by the individuals (time effect: *F* = *44.082, p* < *0.001, ηp*^*2*^ = *0.699*).

The results presented below are reported per outcome variable for the eccentric and concentric contractions respectively.

### Muscle strength

**Figure 3:**
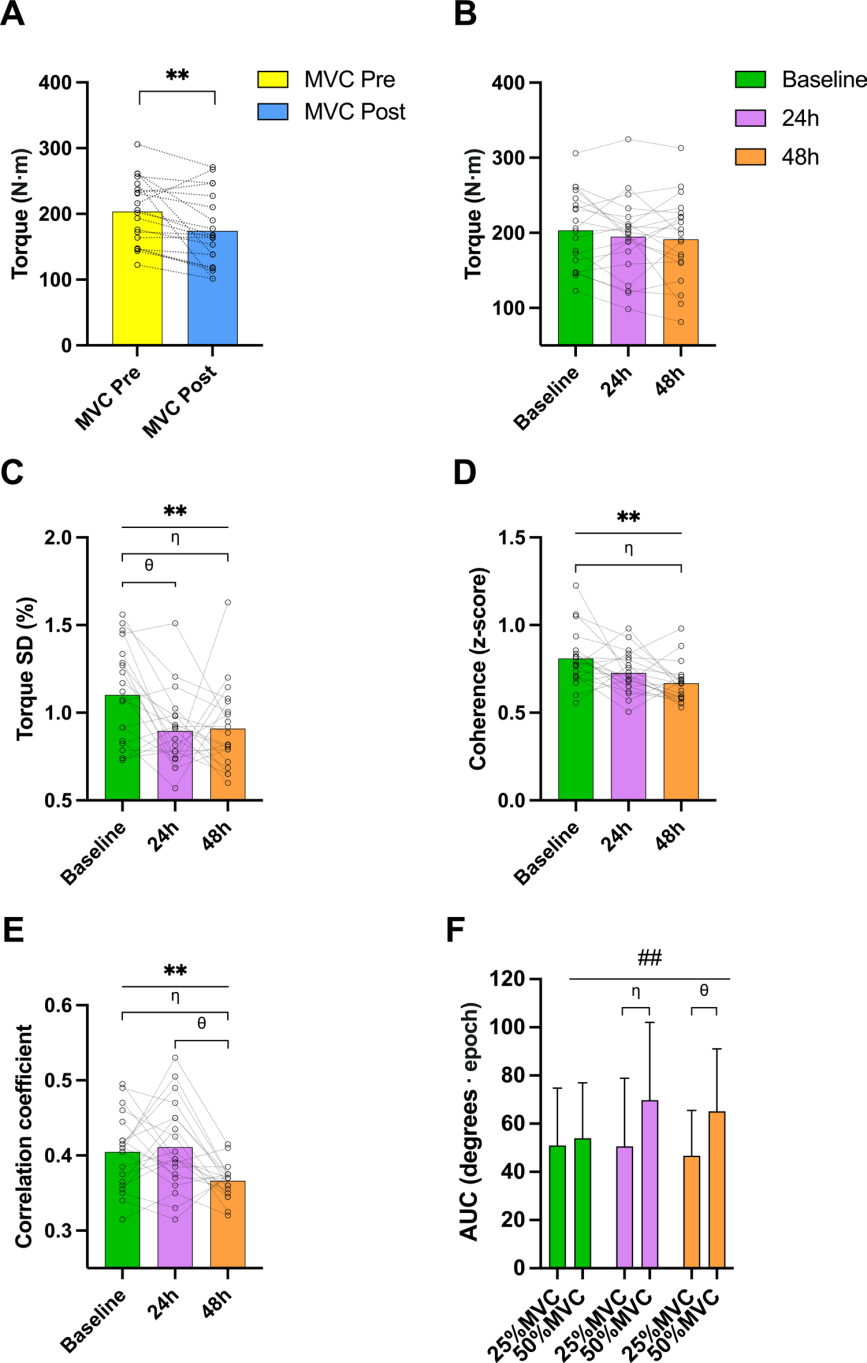
A visual representation of the key results obtained from the eccentric trunk extensions. Eccentric strength during the first session, before and after the exercise protocol to induce delayed onset of trunk muscle soreness (**A**), eccentric strength (**B**), torque SD (**C**), coherence z-scores in the δ band (**D**), HDsEMG-torque cross correlation (**E**) across all three measurement points, area under the curve in the sagittal plane across all measurement points and for both force levels (**F**). The results for graph (**F**) are presented as mean ± SD and for the rest as mean and individual values. For graphs (**B**), (**C**), (**D**) and (**E**) data is pooled and presented across force levels (i.e., 25% and 50%). Main effect of session, *p* < *0.01*: **; session × torque interaction, *p* < *0.01*: ##; *η, θ*: post hoc pairwise comparisons with Bonferroni correction.

In session one, following the eccentric exercise protocol, there was a decrease in peak eccentric torque (i.e., MVC), equivalent to a 29.2 N⋅m or 14.35% decrease (*t* = *3.032*, *p* = *0.007*; Fig. 3A). No decline in peak eccentric torque was observed 24 h and 48 h compared to baseline at the beginning of the session (session effect: *F* = *0.939, p* = *0.379, ηp*^*2*^ = *0.047*; Fig. 3B).

**Figure 4:**
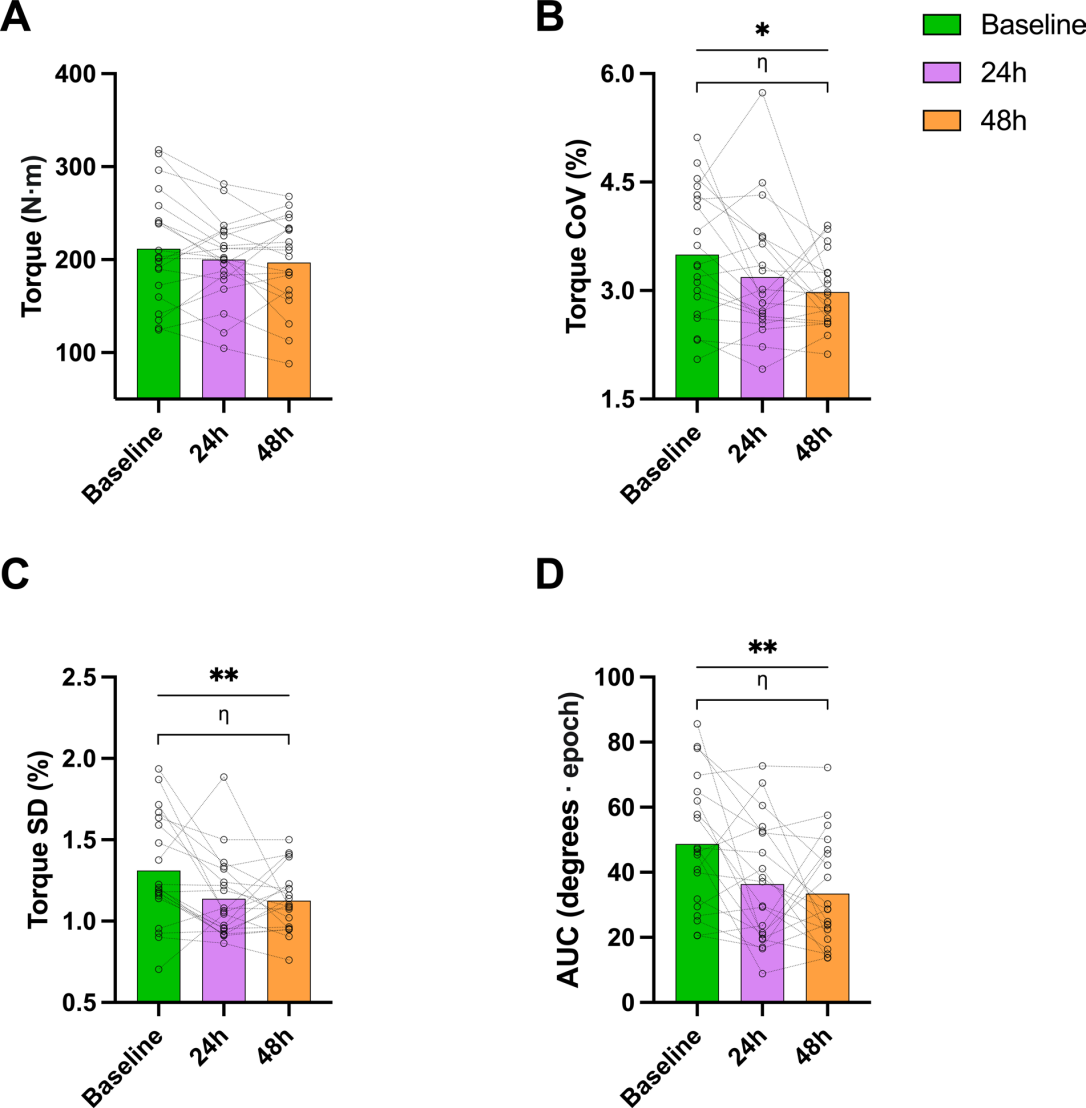
A visual representation of the key results obtained from the concentric trunk extension contractions. Concentric strength (**A**), torque CoV (**B**), torque SD (**C**) over the three measurement points, area under the curve in the sagittal plane across all measurement points and for both force levels (**D**). The results for all graphs are presented as mean and individual values. For all graphs data is pooled and presented across force levels (i.e., 25% and 50%). Main effect of session, *p* < 0.05: *, *p* < 0.01: **; η: post hoc pairwise comparisons with Bonferroni correction.

No decline in peak concentric torque was observed 24 h and 48 h compared to baseline, suggesting that individuals were able to produce the same amount of peak torque during all three sessions irrespective of their muscle soreness and increased mean thoracolumbar muscle sensitivity (i.e., reduced PTT values) (session effect: *F* = *1.896, p* = *0.164, ηp*^*2*^ = *0.091*; Fig. 4A). Mean ± SD peak torque values for both contractions are reported in Table 1.

### Torque steadiness

#### Eccentric contractions

A significant lower torque SD was observed 24 h and 48 h compared to baseline, suggesting that individuals had better torque steadiness in sessions two and three (session effect: *F* = *5.952, p* = *0.006, ηp*^*2*^ = *0.239*; Fig. 3C; baseline: 1.1 ± 0.3%; 24 h: 0.9 ± 0.2%; 48 h: 0.9 ± 0.2%). No differences were observed across sessions for torque CoV (session effect: *F* = *1.295, p* = *0.280, ηp*^*2*^ = *0.263*; baseline: 3.2 ± 0.9%; 24 h: 2.6 ± 0.7%; 48 h: 3.0 ± 1.6%).

#### Concentric contractions

The torque steadiness performance of the participants was better during session 3 compared to baseline as shown from both the torque CoV and SD variables (session effect: *F* = *4.327, p* = *0.020, ηp*^*2*^ = *0.185*; baseline: 3.5 ± 0.9%; 24 h: 3.2 ± 0.9%; 48 h: 3.0 ± 0.5% and *F* = *5.452, p* = *0.008, ηp*^*2*^ = *0.223*; baseline: 1.3 ± 0.3%; 24 h: 1.1 ± 0.3%; 48 h: 1.1 ± 0.2%*,* respectively; Figs. 4B, 3C).

### Electromyographic activity

#### Eccentric contractions

No differences in the level of activation of the thoracolumbar ES were observed across the three sessions (session effect: *F* = *1.250, p* = *0.289, ηp*^*2*^ = *0.062*; baseline: 41.8 ± 21.5 μV; 24 h: 40.6 ± 21.7 μV; 48 h: 38.6 ± 19.7 μV)*.* The regional activation of the thoracolumbar ES along the y- and x-axes was similar during all sessions (session effect: *F* = *0.479, p* = *0.539, ηp*^*2*^ = *0.025*; baseline: 93.7 ± 11.1 mm; 24 h: 95.9 ± 5.5 mm; 48 h: 95.6 ± 6.8 mm and *F* = *1.945, p* = *0.157, ηp*^*2*^ = *0.093*; baseline: 16.2 ± 1.2 mm; 24 h: 16.2 ± 0.9 mm; 48 h: 15.8 ± 1.0 mm).

#### Concentric contractions

No differences in the level of thoracolumbar ES activity were observed across days (*F* = *0.021, p* = *0.923, ηp*^*2*^ = *0.001*; baseline: 44.7 ± 25.1 μV; 24 h: 44.2 ± 21.8 μV; 48 h: 44.2 ± 21.2 μV). The regional activation of the thoracolumbar ES along the y- and x-axes was similar for all three days (*F* = *0.281, p* = *0.656, ηp*^*2*^ = *0.015*; baseline: 96.4 ± 9.5 mm; 24 h: 97.1 ± 8.1 mm; 48 h: 95.2 ± 9.1 mm and *F* = *1.120, p* = *0.337, ηp*^*2*^ = *0.056*; baseline: 16.3 ± 1.0 mm; 24 h: 16.1 ± 0.8 mm; 48 h: 15.9 ± 0.8 mm).

### EMG-torque relationships

#### Eccentric contractions

A session effect was observed for the magnitude of δ band coherence, showing that δ band coherence at 48 h was lower compared to baseline (session 1) and 24 h (session 2) (*F* = *6.685, p* = *0.003, ηp*^*2*^ = *0.260*; Z-coherence in the δ band, baseline: 0.8 ± 0.2; 24 h: 0.7 ± 0.1; 48 h: 0.7 ± 0.1; Fig. 3D). In terms of differences in the topographical representation of δ band coherence along the y- and x-axes, no differences were observed (session effect: *F* = *1.306, p* = *0.276, ηp*^*2*^ = *0.064*; baseline: 94.2 ± 8.1 mm; 24 h: 96.6 ± 2.3 mm; 48 h: 96.1 ± 3.0 mm *and F* = *0.958, p* = *0.393, ηp*^*2*^ = *0.048*; baseline: 16.7 ± 0.5 mm; 24 h: 16.6 ± 0.4 mm; 48 h: 16.5 ± 0.3 mm).

Similarly, a session effect was observed for the magnitude of HDsEMG-torque cross-correlation, showing a reduction in the contribution of the thoracolumbar ES at session 3, compared to the other two sessions (*F* = *7.416, p* = *0.002, ηp*^*2*^ = *0.281*; cross-correlation coefficient, baseline: 0.4 ± 0.0; 24 h: 0.4 ± 0.1; 48 h: 0.37 ± 0.0; Fig. 3E). No differences in the topographical representation of HDsEMG-torque cross-correlation along the y- and x-axes, were observed (session effect: *F* = *0.096, p* = *0.847, ηp*^*2*^ = *0.005*; baseline: 94.3 ± 7.9 mm; 24 h: 94.8 ± 3.2 mm; 48 h: 95.0 ± 4.2 mm *and F* = *1.541, p* = *0.227, ηp*^*2*^ = *0.075*; baseline: 16.7 ± 0.5 mm; 24 h: 16.6 ± 0.4 mm; 48 h: 16.5 ± 0.3 mm).

#### Concentric contractions

No differences were observed for the magnitude of δ band coherence, across days (*F* = *0.373, p* = *0.691, ηp*^*2*^ = *0.019*; Z-coherence in the δ band, baseline: 0.8 ± 0.1; 24 h: 0.8 ± 0.1; 48 h: 0.8 ± 0.1). Similarly, no differences in the topographical representation of δ band coherence along the y- and x-axes, were observed (session effect: *F* = *1.216, p* = *0.297, ηp*^*2*^ = *0.060*; baseline: 94.7 ± 10.4 mm; 24 h: 98.3 ± 5.7 mm; 48 h: 96.8 ± 3.2 mm and *F* = *0.270, p* = *0.765, ηp*^*2*^ = *0.014*; baseline: 16.6 ± 0.4 mm; 24 h: 16.6 ± 0.3 mm; 48 h: 16.5 ± 0.3 mm).

The magnitude of HDsEMG-torque cross-correlation was similar across sessions (*F* = *0.343, p* = *0.712, ηp*^*2*^ = *0.018*; cross-correlation coefficient, baseline: 0.4 ± 0.1; 24 h: 0.4 ± 0.1; 48 h: 0.4 ± 0.1). No differences in the topographical representation of HDsEMG-torque cross-correlation along the y- and x-axes, were observed (session effect: *F* = *1.461, p* = *0.245, ηp*^*2*^ = *0.071*; baseline: 93.7 ± 10.1 mm; 24 h: 97.1 ± 4.8 mm; 48 h: 96.5 ± 3.1 mm *and F* = *0.742, p* = *0.483, ηp*^*2*^ = *0.038*; baseline: 16.6 ± 0.4 mm; 24 h: 16.6 ± 0.3 mm; 48 h: 16.5 ± 0.4 mm).

### Thoracolumbar kinematics

#### Eccentric contractions

A session × torque interaction was observed for the AUC variable in the sagittal plane, suggesting that in sessions two and three, as the task was more demanding (i.e., higher %MVC), individuals increased their trunk flexion ROM (*F* = *6.479, p* = *0.004, ηp*^*2*^ = *0.254*; 25%MVC-24 h: 50.6 ± 28.3°⋅ epoch, 50%MVC-24 h: 69.8 ± 32.2°⋅ epoch; 25%MVC-48 h: 46.7 ± 18.8°⋅ epoch, 50%MVC-48 h: 65.1 ± 25.9°⋅ epoch ;Fig. 3F). Additionally, this interaction showed that at high forces there was a significant difference between sessions°ne and two for this variable (50%MVC-baseline: 54.0 ± 22.9°⋅ epoch, 50%MVC-24 h: 69.8 ± 32.2°⋅ epoch). No significant differences were observed for this kinematic variable nor for the frontal or transverse planes (*p* > *0.05*).

#### Concentric contractions

In Session 3, there was a reduction in the AUC in the sagittal plane compared to the baseline, suggesting decreased thoracolumbar ROM (session effect: *F* = *5.723, p* = *0.007, ηp*^*2*^ = *0.23*; baseline: 48.7 ± 20.0°⋅ epoch; 24 h: 36.4 ± 18.7°⋅ epoch; 48 h: 33.4 ± 16.7°⋅ epoch; Fig. 4D). This might indicate that participants were aiming to maintain a more neutral or stable lumbar spine position. No significant differences were observed for movement in the frontal or transverse planes (*p* > *0.05*).

## Discussion

This study examined the influence of DOMS on torque steadiness, HDsEMG-torque relationships from the thoracolumbar ES, and kinematic data from the thoracolumbar spine in asymptomatic individuals during submaximal concentric and eccentric trunk extension contractions at 25% and 50%MVC. No significant changes were observed for eccentric and concentric trunk extension muscle strength across sessions. The participants demonstrated improved torque steadiness during submaximal concentric and eccentric trunk extension contractions in the presence of DOMS. However, HDsEMG-torque relationships and kinematics were altered in a contraction-dependent manner. During eccentric contractions, a decrease in the thoracolumbar ES contribution to the resultant torque was observed, and individuals showed increased lumbar flexion during the more demanding contractions (50%MVC). During concentric contractions, a reduction in thoracolumbar ROM in the sagittal plane was observed, suggesting the maintenance of a more neutral lumbar spine posture, but no alterations in HDsEMG-torque relationships were observed in the presence of DOMS.

### Muscle soreness and sensitivity

The increased soreness and mean thoracolumbar pressure pain sensitivity observed after 24 h and 48 h support the occurrence of DOMS following the eccentric exercise protocol. This aligns with the observations of previous studies^12,13,38^. Notably, the mild soreness levels we observed at 24 h and 48 h (VAS scores of 2.6 ± 2.1 and 2.9 ± 1.8, respectively) fall within the range of peak soreness levels (2–2.9/10 on the VAS) documented in similar timeframes by other researchers^12^. The observed decrease in mean PPT values aligns with findings from Abboud et al. 2019^38^, who also reported reduced PPT values over the L2-L5 region with the presence of DOMS, suggesting a link to peripheral sensitization driven by inflammatory processes or tissue damage associated with DOMS.

The mechanisms behind DOMS are multifaceted and not fully understood^39^. Traditionally, eccentric exercise-induced microstructural muscle damage was believed to trigger inflammation followed by biochemical, thermal, and mechanical changes, sensitizing muscle afferents and causing soreness and mechanical hyperalgesia^39^. However, recent studies highlight bradykinin, nerve growth factor (NGF), and COX-2-glial cell line-derived neurotrophic factor (GDNF) as key contributors, suggesting that myofiber micro-damage may not be necessary for initiating inflammation or DOMS^39–41^. These molecules could stimulate muscle nociceptors or extracellular receptor binding, indicating their role in mechanical hyperalgesia and inflammation in the extracellular matrix, even without apparent muscle damage^42^. Interestingly, an ultrasound study also suggested an involvement of the paraspinal extramuscular connective tissue (ECT) of trunk extensors in the genesis of DOMS^43^. However, it is important to note that research on these mechanisms has predominantly focused on limb muscles, leaving other areas, such as the trunk, less explored.

### Trunk muscle strength

DOMS typically induces a temporary decrease in muscle force. As expected, following the eccentric exercise protocol, we observed an immediate reduction in eccentric extension trunk strength, which can most likely be attributed to acute muscular fatigue. However, contrary to some prior studies^8,12,30,38^, this decrease in trunk extension MVC was not observed at 24 h and 48 h post-exercise. Reductions in muscle force following eccentric exercise are well-documented for upper and lower limb muscles^8^, however, this trend does not appear to apply as consistently to trunk muscles, particularly in the context of dynamic trunk MVCs. This could be because trunk extension is a multi-joint movement involving the lumbar spine, pelvis, and hips, where potential compensation by lower limb muscles, such as the gluteus maximus and hamstrings, can play a significant role. These muscles can influence both the hip and pelvis, thereby minimizing the engagement of the thoracolumbar extensors. Despite stabilization efforts, such as using straps to secure the thighs and pelvis, it is challenging to prevent these compensatory strategies completely. This suggests that participants might have executed the MVCs by compensating with their hip extensor muscles, particularly if DOMS influenced the use of the thoracolumbar extensors. Furthermore, it is important to consider that participants could also have compensated with upper thoracic muscles of the erector spinae group, whose activity could not be captured with the current electrode placement (e.g., longissimus thoracis and iliocostalis thoracis)^44^. Additionally, contrasting findings may result from protocol differences: (i) previous studies focused on isometric strength, whereas we assessed eccentric/concentric strength, which could increase the likelihood of compensatory torque exertion as mentioned above, (ii) variations in exercise protocol speed (to induce DOMS), load and the use of an isokinetic dynamometer, considering that fast velocity eccentric exercise causes more muscle fibre damage than slow contractions^7^ and because muscle loading level impacts damage extent and recovery rates^10^. Lastly, as mentioned above, individuals might exhibit DOMS without damaging contractile structures, suggesting the involvement of neurotrophic factors and/or connective tissue damage in the increased soreness experienced after exercise^40,41^. These factors likely explain how individuals’ trunk strength was maintained 24 h and 48 h post-exercise.

### Torque steadiness

Considering that DOMS can impair motor function and that torque steadiness is commonly reduced in individuals with CLBP^22,24^, we anticipated a reduction in both eccentric and concentric trunk extension torque steadiness due to DOMS. However, contrary to our hypothesis, torque steadiness improved in both concentric and eccentric contractions 48 h after the eccentric exercise protocol that was used to induce DOMS.

According to previous studies there is conflicting evidence on how DOMS influences the control of muscle force. Some studies^2,45,46^ have observed worse motor control in the presence of DOMS, while others have observed no changes^47^. However, no previous study has assessed trunk extensor torque steadiness in the presence of DOMS. To date, only one study has investigated this aspect using an alternative model of acute experimental pain. Specifically, this study found no significant changes in trunk extensor force variability following intramuscular injection of hypertonic saline into the longissimus muscle^48^. In contrast, investigations into lower/upper limb muscles^9^, observed significant increases in force fluctuations, but only immediately and 1 h after eccentric exercise, with no differences observed at 24 h and 48 h. Similarly, a study investigating the knee extensors^46^ observed that force steadiness deficits were more pronounced immediately post-exercise and primarily at lower %MVC levels (2.5, 5 and 10%), with less impact at 20 or 30% MVC, and no data available for periods beyond 24 h or for higher loads.

Collectively, the findings from these studies support our observation that DOMS does not necessarily lead to reduced force steadiness. Additionally, it is important to consider that immediate reductions in force steadiness, observed by some studies^9,46^, are mainly attributed to acute muscular fatigue following eccentric exercise rather than DOMS which occurs later. The lack of reductions in force steadiness in the presence of trunk extensor DOMS can be attributed to some factors, including: (i) compensatory mechanisms inherent in multi-joint movements, such as trunk extension, which allow for significant compensatory strategies when experiencing DOMS. The activation of the hamstrings, gluteals and the not recorded parts of the thoracic ES to compensate for decreased functionality in the sore thoracolumbar ES and (ii) a learning effect, possibly linked to the improved force steadiness after 48 h. This effect, likely arising from frequent task repetition with visual feedback during the eccentric exercise protocol, demonstrates that despite the presence of DOMS or acute discomfort, individuals can enhance their performance, suggesting this sensation does not necessarily hinder the learning and adaptation process. This notion is further supported by a previous study^49^ using an experimental pain model, where individuals with acute shoulder pain showed an improvement level in movement accuracy during fast arm-reaching movements and force field perturbations comparable to that of pain-free controls. Furthermore, this improvement in force steadiness may also be understood as a strategy for individuals to maintain a more consistent force output, thereby avoiding increases in the sensation of discomfort that may result from a less steady force output with sudden alterations. Additionally, the use of HDsEMG and kinematic data in the current study helped explore these improvements further.

### HDsEMG amplitude and HDsEMG regional activation

During both eccentric and concentric contractions, we observed no changes in the magnitude of thoracolumbar ES activation or regional activity in the presence of DOMS. These findings align with similar previous research on the trunk extensors^12,30^ and medial gastrocnemius^50^, further supporting the theory related to the involvement of muscle-associated connective tissue^39,51^ instead of changes in the muscle itself. A review^3^ has previously aimed to examine the course of EMG changes and alterations at the motor unit level. However, the most common muscle assessed was the biceps brachii, and most differences were reported immediately after an eccentric exercise protocol, 2 h and 24 h. Importantly, it highlighted an inconsistency of change in agonistic and antagonistic EMG amplitude at 24 h, and no data were presented at 48 h. Interestingly, many changes can happen at the motor unit level. However, capturing these changes using HDsEMG in the thoracolumbar ES poses significant challenges. By examining the HDsEMG-torque relationship, we gained additional insights into why individuals show improved torque steadiness in the presence of DOMS. Nevertheless, it is important to note that DOMS is not always indicative of muscle damage, which might explain some of the inconsistency in the findings and the absence of differences in EMG variables when DOMS is present. It is important to mention that most of these changes at a motor unit level were reported immediately after eccentric exercise, thereby not excluding, or underestimating the confounding factor of fatigue in these observations.

### HDsEMG-torque relationships

The magnitude of HDsEMG cross-correlation and δ band coherence at 48 h post-exercise was reduced during eccentric trunk extensions when compared to the previous two sessions. However, no regional changes in δ band coherence maps were observed during either eccentric or concentric contractions at 24 h and 48 h post-exercise. This uniformity in δ band coherence, reinforced by the PPT maps, which revealed no regional sensitivity differences in the thoracolumbar area, suggests that the changes were primarily in magnitude and indicates a highly localised nature of DOMS. Another noteworthy observation was the absence of differences in the magnitude of these variables during concentric trunk extension contractions across days. This is not surprising, considering the inherent differences between eccentric and concentric contractions. Eccentric contractions involve greater passive muscle element contribution, distinct neural control, higher force production with lower metabolic energy consumption, and increased mechanical stress, resulting in more microtrauma^40,52,53^. Consequently, due to the increased stress on connective tissue and muscle fibres during DOMS, we can anticipate more pronounced changes in these patterns, aiding in task adaptation and performance enhancement. Furthermore, during eccentric contractions, individuals are likely to leverage this additional component effectively for torque production. These insights could be further elucidated by examining thoracolumbar kinematics, as detailed in the following section.

### Thoracolumbar kinematics

Distinct differences were observed in the sagittal plane during both eccentric and concentric contractions. Notably, in the presence of DOMS, individuals demonstrated increased lumbar flexion during eccentric contractions at both 24 h and 48 h, particularly at higher loads (i.e., 50%MVC). This pattern was not observed at baseline, suggesting that the individuals had to alter their movement pattern to be able to perform the more demanding trunk extension eccentric contractions. In contrast, during the concentric contractions, different adaptations were observed. At 48 h, individuals used a more neutral or stable lumbar spine position during the concentric contractions compared to baseline. The lack of notable differences in other planes of movement could be attributed to the individual's position on the chair, which was stabilised by straps, and the attachment system that limits movements to the frontal and transverse planes.

Even though distinct, the adaptations observed in movement patterns during both eccentric and concentric contractions could likely serve two functions. Firstly, these adaptations might be a self-protective mechanism for the tissues involved, potentially reducing the risk of further damage. Secondly, they may be related to a learning effect, particularly considering the exercise protocol's similarity with the torque steadiness tasks, enabling more efficient and effective task performance. For example, in the case of eccentric extension, an increased lumbar flexion can be explained in two ways, (i) it might be related to a more efficient utilisation of the passive muscle elements or of the extramuscular connective tissue and/or (ii) it could represent a protective strategy to reduce strain on these tissues by engaging compensatory muscle groups such as the hamstrings, gluteals and/or other parts of the thoracic ES. This explanation is supported by recent findings showing that eccentric exercise can lead to increases in lumbar extramuscular connective tissue thickness^43^ and immediate changes in the optimum length for force generation in the hamstring muscles^6^.

Conversely, the controlled back extension observed during concentric movements could contribute to minimising unnecessary thoracolumbar motions. This controlled motion might optimise the length-tension relationship in the trunk extensor muscles, avoiding overly stretched or contracted positions. Consequently, this approach could reduce the risk of further damage to the connective or muscle tissue and likely enhance force steadiness by maintaining muscle efficiency and stability.

### Methodological considerations

A potential limitation of the study relates to the generalizability of the results, considering the sample primarily consisted of young and highly active individuals. Such a group may exhibit a faster recovery from DOMS compared to older individuals or those with lower levels of physical activity. Despite this, the presence of mechanical hyperalgesia and mild muscle soreness did confirm the presence of DOMS in our sample. Moreover, while the positioning of individuals could have allowed for compensation using hip, gluteal and/or other parts of the thoracic ES muscles during the task, we aimed to minimise such compensatory movements in our protocol. This was achieved by restricting movement with straps over the pelvis and thighs and by instructing participants to rely mainly on their trunk extensor muscles as they performed the task. Another limitation of the study is the use of rectified sEMG to estimate neural drive to muscles. While sEMG signals can be influenced by various factors, the rationale behind choosing this method has been previously detailed ^23,24^. It is also important to acknowledge that factors such as skin temperature, hydration, and electrolyte balances, which can affect sEMG signals, were not controlled in this study. However, the reliability of HDsEMG for these muscles has been previously established^33^, supporting the robustness of our findings.

Another important consideration is the specific load, speed, and measurement time points chosen for this study. Although changes were observed and DOMS was induced by the eccentric exercise protocol, employing an eccentric protocol with maximal effort and faster contractions, along with incorporating immediate and 2-h post-task measurements, might have revealed additional insights, particularly in terms of HDsEMG measures related to thoracolumbar ES activity, as indicated in previous research for limb muscles^3^. Lastly, it would be beneficial to confirm our speculation regarding the contribution of lower limb musculature to the resultant torque and gain insights into the behaviour of synergistic muscles. The positioning of the electrodes, coupled with the seated posture of the participants, restricted our ability to place additional electrodes on these lower limb muscles.

## Conclusion

This study uniquely demonstrates that in the presence of DOMS, individuals exhibit improved torque steadiness during trunk concentric and eccentric extension contractions, likely due to adaptations of movement and muscle recruitment strategies, influenced by a learning effect from initial training exposure. This also shows that contrary to individuals with CLBP who may lack the motor resources to compensate for motor control impairments, resulting in reduced torque steadiness, pain-free individuals can make adjustments that lead to improvements in torque steadiness. Importantly, the study also reveals that movement patterns differ significantly between contractions in the presence of DOMS. Increased lumbar flexion was observed in the more challenging eccentric contractions, whereas in concentric contractions, there was a noticeable reduction in thoracolumbar (sagittal) movement. This variation in behaviour could suggest a strategy of protecting the involved tissues and learned efficiency to optimise torque steadiness performance. Collectively, the findings of this work underscore the significance of adaptive strategies in response to DOMS and their influence on muscular performance.

## Data Availability

The datasets generated and/or analyzed during the current study are available from the lead author (MA) upon reasonable request.
